# Attitudes of Family Medicine Program Directors on Including Race in Clinical Presentations: A Cross-Sectional Survey Study

**DOI:** 10.7759/cureus.100767

**Published:** 2026-01-04

**Authors:** Ivonne McLean, Rachel Rosenberg, Mara Phelan, Angelica Lopez, Ping-Hsin Chen

**Affiliations:** 1 Department of Family Medicine and Community Health, Institute for Family Health, Mount Sinai Hospital, New York, USA; 2 Department of Family Medicine, Rutgers University New Jersey Medical School, Newark, USA

**Keywords:** anti-racism in medical education, clinical skills, health equity, medical education, mitigating implicit biases

## Abstract

Background and objectives

Clinicians’ implicit biases contribute to persistent healthcare disparities in the United States. Including patient race or ethnicity in the opening line of a clinical presentation (e.g., “A 40-year-old Black woman presents with…”) reinforces the false notion of race as a biological construct and can trigger unconscious biases. This study surveyed U.S. Family Medicine Residency program directors (PDs) to assess attitudes toward this practice and to identify factors associated with support or opposition.

Methods

Study questions were included in the 2021 Council of Academic Family Medicine Educational Research Alliance (CERA) omnibus survey of all ACGME-accredited Family Medicine residency PDs, approved by the American Academy of Family Physicians Institutional Review Board in Leawood, Kansas, USA. The primary outcome was the PD's attitude toward including race or ethnicity in the opening line. Data analysis was performed at Rutgers New Jersey Medical School in Newark, New Jersey, USA. Analyses examined associations with PD demographics, program characteristics, and participation in implicit bias training.

Results

Of 631 eligible PDs, 275 responded (response rate: 43.6%). Among the 260 respondents who answered the primary question, 35.8% opposed including race in the opening line, 45.8% supported its use in select circumstances, and 18.5% supported using it in all circumstances. Opposition was more common among PDs identifying as female and among those identifying as American Indian/Alaska Native, Black, or other race. Recent implicit bias training was associated with greater opposition, although not statistically significant.

Conclusions

Many PDs continue to support including race in opening clinical statements despite increased awareness of how such practices may contribute to bias. These findings highlight the need for clear guidance, faculty development, and equity-focused curricular strategies that promote thoughtful and evidence-based approaches to discussing patient identity in clinical education.

## Introduction

Inequities in health outcomes among racial and ethnic groups in the United States remain persistent despite decades of targeted health system efforts to eliminate disparities [1]. Growing evidence demonstrates that clinicians’ implicit biases contribute to these inequities by shaping diagnostic decisions, treatment recommendations, and interpersonal communication [2]. Implicit bias, or unconscious bias, refers to attitudes and beliefs that operate outside of conscious awareness and may not align with an individual’s explicit values [3]. Like all people, healthcare professionals are susceptible to decision-making influenced by these biases, but can also learn to identify, address, and decrease such responses. Recognizing the potential for intervention, the Association of American Medical Colleges (AAMC) and other organizations have advocated for strategies to identify and mitigate implicit bias in clinical care [4].

Unfortunately, some longstanding communication practices in academic medicine may unintentionally contribute to or reinforce biased thinking. One example is the routine inclusion of a patient’s race or ethnicity in the opening line of a clinical presentation (e.g., “Mr. Smith is a 40-year-old Black man who presents with…”). Research shows that this phrasing can reinforce the false notion that race is a biological category and can activate unconscious stereotypes among listeners [5]. In response, several medical associations and other stakeholders in medical education have proposed eliminating the routine inclusion of race in opening clinical statements as a strategy to reduce bias [6].

Despite these recommendations, little is known about how widely this guidance has been adopted in residency training. Program directors (PDs) play a central role in shaping clinical communication norms, modeling presentation practices, and setting expectations for trainees. Clinical communication habits formed early in training often persist throughout a physician’s career. Understanding PDs’ perspectives is therefore essential to identifying opportunities for improvement and ensuring alignment between health equity goals and day-to-day teaching practices.

To address this gap, we surveyed Family Medicine residency PDs to assess their attitudes toward including patient race or ethnicity in the opening line of clinical presentations and to examine factors associated with support or opposition. The primary objective of this study was to assess Family Medicine residency PDs' attitudes toward including race or ethnicity in the opening line of clinical presentations. A secondary objective was to examine whether PD and program characteristics, including demographics, geographic region, faculty characteristics, and experience with implicit bias training, were associated with these attitudes.

This article was previously presented as a poster at the 2023 STFM (Society of Teachers in Family Medicine) Annual Spring Conference.

## Materials and methods

Study design

This was a descriptive, cross-sectional survey study that surveyed PDs of family medicine residency programs in the United States. Study questions were part of a larger omnibus survey conducted by the Council of Academic Family Medicine Educational Research Alliance (CERA) [7] and approved by the American Academy of Family Physicians Institutional Review Board (approval #19-366_A12, dated 9/6/2021). The study was done through the IRB of the American Academy of Family Physicians in Leawood, Kansas, USA. The academic institution where the data analysis was performed was Rutgers New Jersey Medical School in Newark, New Jersey, USA. The methodology of the CERA PD Survey has been described in detail elsewhere [8]. The authors submitted their proposed survey questions to CERA through the competitive item-submission process, where the questions were reviewed for clarity, relevance, and methodological quality. Following acceptance, the items were included in the 2021 CERA PD omnibus survey. After survey administration, CERA provided the authors with the de-identified dataset and granted permission for use of the data in analysis and publication.

Participants and sampling frame

The sampling frame consisted of all ACGME-accredited United States family medicine residency PDs, as identified by the Association of Family Medicine Residency Directors (AFMRD). Eligible participants were PDs from established residency programs, defined as programs with at least three resident classes in place at the time of the survey.

At the time of the survey, 698 PDs were identified. Two had previously opted out of SurveyMonkey® surveys, and 30 email invitations bounced, resulting in 666 successfully delivered invitations. A qualifying survey question assessed program eligibility; PDs from programs that had not yet established three resident classes were excluded (n = 35), yielding a final eligible sample of 631 PDs. A total of 275 PDs completed the survey, resulting in a response rate of 43.6% (275/631).

Survey instrument

The CERA survey instrument included structured multiple-choice questions addressing PD demographics, program characteristics, faculty composition, and the presence of implicit bias or related training. The primary outcome variable assessed PD attitudes toward including patient race or ethnicity in the opening line of a clinical presentation. PDs were asked:

“Which of the following best describes your attitude toward the practice of including patient race/ethnicity in the opening line of a clinical presentation?”

Response options were Do Not Support, Support in Select Circumstances, and Support in All Circumstances.

Data collection

Data were collected electronically using SurveyMonkey® between September 14, 2021, and October 8, 2021. Email invitations were sent to all eligible PDs, followed by three weekly reminders to non-respondents and a final reminder sent two days before survey closure. Participation was voluntary and anonymous.

De-identified response data were subsequently released to the authors by CERA in accordance with established data-access procedures.

Variables

The primary dependent variable was the PD’s selected response to the question regarding support for including race or ethnicity in the opening line of a clinical presentation. Independent variables included: *PD race/ethnicity and self-identified gender, residency program region*, *PD's past participation in implicit bias training*, and* percentage of faculty underrepresented in medicine (URiM) in the residency program.* Missing data for individual variables were treated as missing and excluded from analyses involving those variables.

Statistical analysis

Bivariate analyses were conducted to evaluate associations between attitudes toward including race or ethnicity in the opening line of a clinical presentation and PD or program characteristics. Chi-square tests were used to compare categorical variables. All p-values were generated using chi-square test outputs in IBM SPSS Statistics version 28 (IBM SPSS Statistics version 28.0 (IBM Corp., Armonk, NY)). Statistical significance was defined as a two-sided p-value < 0.05.

## Results

Of the 631 PDs contacted, 275 responded (response rate: 43.6%). Of these, 260 PDs (94.5%) completed the primary question regarding the inclusion of patient race or ethnicity in the opening line of a clinical presentation. Results, organized by respondent characteristics, are summarized in Table 1. 

**Table 1:** Summary: PD attitudes towards utilization of race/ethnicity in opening line of clinical presentation a. Race/ethnicity and gender categories identified by the US Census Bureau 2020. b. The statistical significance was set at p = 0.05. P-values were generated using chi-square analyses. c. Regions: Northeast = New England (NH, MA, ME, VT, RI, or CT) and Middle Atlantic (NY, PA, or NJ); West = Pacific (WA, OR, CA, AK, or HI) and Mountain (MT, ID, WY, NV, UT, AZ, CO, or NM); Midwest = East North Central (WI, MI, OH, IN, or IL) and West North Central (ND, MN, SD, IA, NE, KS, or MO); South = West South Central (OK, AR, LA, or TX),  East South Central (KY, TN, MS, or AL) and South Atlantic = FL, GA, SC, NC, VA, DC, WV, DE, PR, or MD d. AAMC’s 2004 URiM definition includes Black/African-American, Hispanic/Latino/of Spanish Origin, American Indian/ Alaska Native, Native Hawaiian/other Pacific Islander, and other racial and ethnic groups underrepresented in the medical profession relative to their numbers in the general population. e. Implicit bias training was defined as “a curriculum to help clinicians identify and mitigate their own implicit biases.” f. “Valid n” refers to the number of cases with a valid response for the particular variable.

	Which of the following best describes your attitude toward the practice of including patient race/ethnicity in the opening line of a clinical presentation? (e.g., "Ms. Smith is a 40 year old Black woman who presents with...")				
	Do not support	Support in select circumstances	Support in all circumstances	N (total n = 260)	p-value^b^
PD self-identified gender^a^				Valid n^f^ = 259	p = 0.006
Female	55 (46.2%)	49 (41.2%)	15 (12.6%)	119 (100%)	
Male	36 (26.5%)	68 (50%)	32 (23.5%)	136 (100%)	
Other/non-binary	2 (66.7%)	1 (33.3%)	0 (0.0%)	3 (100%)	
Choose not to disclose	0 (0.0%)	0 (0.0%)	1 (100%)	1 (100%)	
PD self-identified race				Valid n = 259	p = 0.011
American Indian/Alaska Native	3 (100%)	0 (0%)	0 (0%)	3 (100%)	
Asian	4 (16%)	13 (52%)	8 (32%)	25 (100%)	
Black/ African American	7 (63.6%)	2 (18.2%)	2 (18.2%)	11 (100%)	
White	76 (37.1%)	95 (46.3%)	34 (16.6%)	205 (100%)	
Other	3 (100%)	0 (0%)	0 (0%)	3 (100%)	
Choose not to disclose	1 (8.3%)	7 (58.3%)	4 (33.3%)	12 (100%)	
PD self-identified ethnicity				Valid n = 253	p = 0.327
Hispanic/Latino	6 (30%)	8 (40%)	6 (30%)	20 (100%)	
Non-Hispanic/Latino	87 (37.3%)	107 (45.9%)	39 (16.7%)	233 (100%)	
Residency region^c^				Valid n = 260	p = 0.002
Northeast	24 (47.1%)	21 (41.2%)	6 (11.8%)	51 (100%)	
South	12 (16.2%)	42 (56.8%)	20 (27%)	74 (100%)	
Midwest	33 (47.1%)	25 (35.7%)	12 (17.1%)	70 (100%)	
West	24 (36.9%)	31 (47.7%)	10 (15.4%)	65 (100%)	
%Faculty underrepresented in medicine (URiM)^d^				Valid n = 257	p = 0.159
<20%	63 (35.8%)	85 (48.3%)	28 (15.9%)	176 (100%)	
21-50%	24 (40.7%)	23 (39%)	12 (20.3%)	59 (100%)	
51-80%	4 (25%)	7 (43.8%)	5 (31.3%)	16 (100%)	
81-100%	0 (0%)	3 (50%)	3 (50%)	6 (100%)	
Implicit bias training^e^				Valid n = 260	p = 0.066
Yes, in the past 12 months	76 (42%)	77 (42.5%)	28 (15.5%)	181 (100%)	
Yes, more than 12 months ago	11 (22%)	28 (56%)	11 (22%)	50 (100%)	
No	4 (20%)	10 (50%)	6 (30%)	20 (100%)	
Not sure	2 (22.2%)	4 (44.4%)	3 (33.3%)	9 (100%)	

**Figure 1:**
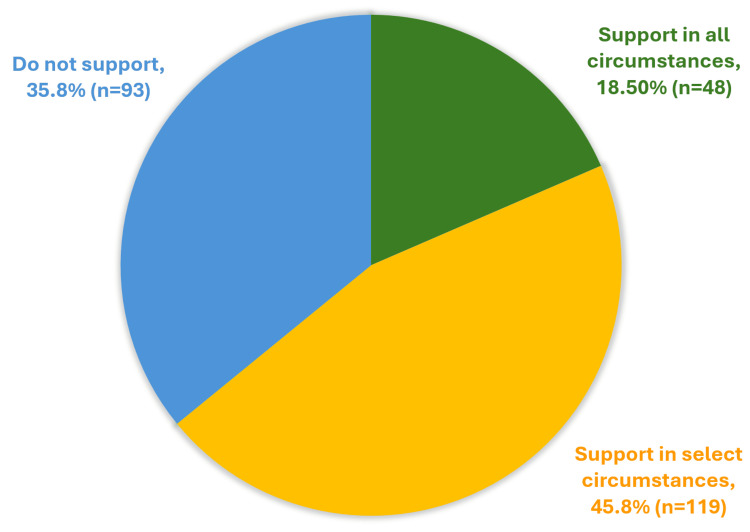
Program director attitudes toward including race/ethnicity in the opening line of clinical presentations

Among these 260 PDs, 35.8% (n = 93) opposed the practice, 45.8% (n = 119) supported it in select circumstances, and 18.5% (n = 48) supported it in all circumstances (Figure 1).

Differences by gender identity

Attitudes toward the inclusion of race differed significantly by PD gender (p = 0.006). PDs who identified as female were significantly more likely to oppose the practice in all circumstances (n = 55, 46.2%) compared with male PDs (n = 36, 26.5%). Among PDs identifying as non-binary or other, two of three (n=2, 66.7%) also opposed the practice.

Differences by race/ethnicity

Significant variation was observed by PD race (p = 0.011). A majority of Black PDs (n = 7, 63.6%) opposed the practice of including race in the opening line, compared to 37.1% (n = 76) of White PDs and 16% (n = 4) of Asian PDs. All respondents identifying as American Indian/Alaska Native (n = 3) and those identifying as “Other” (n = 3) opposed the practice. No statistically significant difference was found by Hispanic/Latino/a/x ethnicity (p = 0.327).

Differences by region

Statistically significant findings (p = 0.002) were also noted based on regions of the country. PDs in the Northeast (n = 24, 47.1%) and Midwest (n = 33, 47.1%) were more likely to oppose the practice of including race in the opening line of the clinical presentation, whereas PDs in the South (n = 42, 56.8%) and West (n = 31, 47.7%) were more likely to support it in select circumstances.

Implicit bias training

PDs who had received implicit bias training within the past 12 months were more likely to oppose including race in the opening line (n = 76, 42%) compared to those trained more than 12 months ago (n = 11, 22%) or not at all (n = 4, 20%). While this trend was not statistically significant when analyzed by training status alone (p = 0.066), significance was reached when responses were grouped into “any support” versus “no support” for the practice (p = 0.018).

No significant associations were observed based on the percentage of faculty underrepresented in medicine (URiM) at the PDs’ institutions (p = 0.159).

## Discussion

In this national survey of Family Medicine residency PDs, we found substantial variation in attitudes toward including race in the opening line of clinical case presentations. While many respondents indicated alignment with emerging recommendations to avoid routine racial framing in clinical communication [9], a majority of respondents still supported including race under certain circumstances. These patterns are consistent with prior work showing that race continues to be used as a clinical heuristic, even as medical education increasingly emphasizes race as a social rather than biological construct [10].

Our findings also suggest that implicit bias training alone may not be sufficient to shift entrenched communication habits. PDs who reported recent participation in implicit bias training did not differ markedly from those without such training, which aligns with literature showing that single-session or didactic-only interventions may have limited impact on practice change [11]. These findings underscore the need for continued faculty development and clear institutional guidance to ensure alignment between health equity goals and day-to-day teaching practices.

Strengths and limitations

A major strength of this study is its use of a national sampling frame of Family Medicine residency PDs, allowing perspectives from diverse training settings across the United States. The response rate of 43.6% is comparable with other national surveys of residency leadership [12], increasing confidence in the representativeness of our findings. In addition, the survey items were developed through the established CERA question-submission and review process, which supports content clarity and relevance.

This study also has important limitations. Responses reflected self-reported attitudes, which may be influenced by social desirability bias, given the sensitive nature of topics related to race and bias. The cross-sectional design captures attitudes at a single point in time, limiting inferences about trends over time. Participation was voluntary, and it is possible that PDs with a greater interest in issues of equity or communication were more likely to respond. The survey assessed beliefs rather than behaviors, and PDs’ reported attitudes may not reflect actual teaching practices or trainee experiences. Additional limitations include limited generalizability beyond Family Medicine PDs, the potential for nonresponse bias, and small sample sizes within some demographic subgroups. The primary outcome was assessed using a single survey item, which may not capture the full nuance of PDs’ communication practices. Finally, only bivariate analyses were conducted, limiting the ability to explore multivariable relationships or causal pathways.

Future directions and recommendations

Future research should examine how race is used in real clinical teaching, including direct observation of learner presentations and faculty feedback. Studies that evaluate institutional strategies such as standardized case-presentation language, structured equity curricula, and faculty coaching will help determine which approaches most effectively shift long-standing habits. Longitudinal research could assess whether program-level interventions lead to durable changes in faculty practices and learner communication skills.

Professional organizations and accreditation bodies may play an important role by offering standardized guidance, case-presentation templates, and examples of best practices that reduce reliance on race-as-biology frameworks. Residency programs can also support change by explicitly teaching structural competency [13], embedding expectations into case-presentation frameworks, offering consistent faculty development, and integrating equity-focused competencies into assessment systems. Continued work is needed to ensure that communication practices reflect an evidence-based, patient-centered, and equity-oriented approach to discussing patient identity and context.

## Conclusions

This study demonstrates meaningful variation in how Family Medicine residency PDs view the use of race in the opening line of clinical case presentations. While many respondents were in alignment with current recommendations by opposing the practice, a substantial proportion continued to endorse including race under certain or all circumstances, suggesting that longstanding communication habits remain influential in clinical teaching. Concerns about this practice stem from its potential to reinforce race as a biological construct and to bias the listener by activating unconscious stereotypes that may influence clinical reasoning and decision-making. PDs who reported participation in implicit bias training did not consistently differ in their attitudes from those without such training, indicating that isolated or didactic interventions alone may be insufficient to shift entrenched norms; however, given the cross-sectional design, these findings should not be interpreted as evidence of causal effects. Together, these results underscore the need for clear guidance, targeted faculty development, and structured curricular support to promote communication practices that reflect contemporary understandings of race as a social rather than biological construct. As educators work to prepare trainees for equitable and patient-centered care, structured approaches to case presentation that emphasize social and structural context may help align teaching practices with these goals, and future research, including observational studies of teaching practices and longitudinal evaluations of educational interventions, will be essential to identifying strategies that meaningfully advance clinical communication and promote more equitable care delivery.
